# An anatomical study of arcuate foramen and its clinical implications: a case report

**DOI:** 10.1186/s12998-016-0082-2

**Published:** 2016-01-25

**Authors:** Salman Afsharpour, Kathryn T. Hoiriis, R. Bruce Fox, Samuel Demons

**Affiliations:** Basic Science Division, Department of Anatomy, Life University, College of Chiropractic, 1269 Barclay Circle, Marietta, GA 30060 USA; Chiropractic Sciences Division, Life University, College of Chiropractic, 1269 Barclay Circle, Marietta, GA ᅟUSA; Clinical Sciences Division, Department of Radiology, Life University, College of Chiropractic, 1269 Barclay Circle, Marietta, GA ᅟUSA

**Keywords:** Arcuate foramen, C1 dorsal arch, Vertebral artery

## Abstract

**Background:**

The objective of this paper is to describe the relationship of the vertebral artery (VA) to the Atlas (C1) in the sub-occipital region in the presence of arcuate foramen; and discuss the clinical implications related to manual therapies and surgical implications related to screw placement. This study is an anatomical cadaveric case report of symmetrical bilateral lateral and dorsal arcuate foramina on the C1 dorsal arch.

**Case Presentation:**

Out of 40 cadavers that were available for use in teaching anatomy in the university setting, three presented with anomalies of the C1 dorsal arch. The sub-occipital regions were skillfully prosected to preserve related structures, especially VAs, sub-occipital and greater occipital nerves. Visual observations, photographs, measurements, and radiographic examinations were performed between January 15, 2014 and August 25, 2014. One cadaver (Specimen A) presented with complete bilateral ossified arcuate foramina, and two presented with partial ossification of the atlanto-occipital membrane. Specimen A presented the bilateral anomaly which is almost symmetrical. The VAs were found passing through double foramina (lateral and dorsal) on each side.

**Conclusions:**

Arcuate foramina have been shown to be commonly found anomalies with highly variable shapes and sizes, even in the same individual with a bilateral condition. This study found a rare type of the anomaly associated with the C1 dorsal arch, which protected the VA against manual pressure. However, VA, in this case, would be more susceptible to dissection. The presence of the arcuate foramen would also complicate screw placement during surgery. Clinical pre-screening for signs of vertebrobasilar insufficiency is important for chiropractic and manual therapies.

## Background

The Atlas, located at the cranio-cervical junction, is a ring-shaped vertebra. Normally, the vertebral artery glides easily with neck movements as it lies in the groove at the supero-lateral aspect of the C1 dorsal arch. The relationship of the VA to the dorsal arch of C1 has been well described. Cacciola, Ebraheim, and others have studied the course of the artery and the parameters relevant during surgery in the region [1–8]. Cacciola, et al. injected colored silicone into the arteries and veins of ten cadaveric specimen; and the microsurgical anatomy of the VAs were evaluated along its course from the C3 transverse process to its entrance into the vertebral foramen at the occipito-atlantal (C0-C1) level with particularly close inspection of the relationship to the C2 vertebra [1]. The authors concluded that the intimate relationship makes the VA susceptible to injury during the surgical procedures in the region [1]. The multiple loops of the artery provide VA extra length which is probably essential to avoid any stretch during neck movements [1].

Certain anomalies of the Atlas vertebra may have clinical significance for surgery, chiropractic and other manual therapies [9–14]. Arcuate foramen have been shown to be commonly found anomalies with highly variable shapes and sizes [1–8, 15, 16]. A bony bridge is formed by ossification in the oblique part of the atlanto-occipital membrane above the passage of VA [15]. There are several names used for the bony bridge, among them: posterior ponticle, posticus ponticus, ponticulus posticus, kimmerle anomaly or arcuate foramen [15]. Of grave concern is whether the posterolateral bony bridge could involve VA compression and/or fixation (tethering). Cushing et al. reported that presence of arcuate foramen caused increased incidence of VA dissection because of tethering as it passed through the osseous bridges of arcuate foramina [9]. The number of patients treated with C1 lateral mass screws through the posterior arch has dramatically increased recently, therefore it is important to recognize the presence of arcuate foramen before performing the Goel procedure for placement of the screws [15–19].

A meta-analysis, by Elliot and Tanweer, demonstrated that arcuate foramen anomalies are not rare [15]. Their report described a systematic review of radiographic, cadaveric, and surgical data. They reported overall prevalence of arcuate foramina was 16.7 %; with 18.8 % in cadaver studies, 17.2 % in computed tomography studies, and 16.6 % in x-ray studies. Standard radiographs cannot demonstrate bilateral versus unilateral arcuate foramina. Elliot and Tanweer reported complete foramen in 9.3 % of patients and a partial or incomplete foramen in 8.7 %. In 5.4 % of cases, complete foramina were present bilaterally. In 7.6 % of cases, it was unilateral. They found no difference in prevalence between males and females [15].

Measurements of arcuate foramina are of interest because of the highly variable nature of the anomalies and their clinical implications. The arcuate foramina may possibly compress the VA. In a study by Krishnamurthy et al., 1044 complete undamaged dry human atlas vertebrae were examined [13]. These researchers found the trait was present in 13.8 % of their samples. They measured the mean length of the arcuate foramen at 7.16 mm on the left side and 9.99 mm on the right side in bilateral positive samples. It was 8.14 mm and 9.26 mm respectively in unilateral positive samples. They reported mean vertical height of arcuate foramen was 6.57 mm on the left side and 6.52 mm on the right side in bilateral positive samples. It was 4.91 mm and 5.38 mm respectively in unilateral positive samples. Besides genetic factors, the researchers discuss that mechanical external factors, such as carrying heavy objects on the head, could also play a role in the development of bridges [13]. It has been suggested that healthcare providers, including neurologists, neurosurgeons, and the medical community, in general, should have knowledge about the present variation and should try to look for it when dealing with the patients complaining of symptoms of vertebrobasilar insufficiency like headache, vertigo, and shoulder and arm pain [9–13, 14, 20–22].

This report describes an unusual finding during anatomy course instruction with cadavers provided by the Life University, College of Chiropractic. Anatomists generally recognized that skillful prosection leads to better visualization, demonstration and description for student learning [23, 24]. Anatomical descriptions of location, relation to neighboring structures, size and shape are often supported by drawings, but not often by photographic or radiographic images [23]. An increase in the use of computers for teaching anatomy has been reported [24]. Our study provided photography and x-rays of this clinically important anomaly for use in teaching. This report will discuss the anomalies of arcuate foramen found and clinical significance for surgery, as well as chiropractic and other manual therapies.

## Case presentation

### Methods and materials

**Fig. 1:**
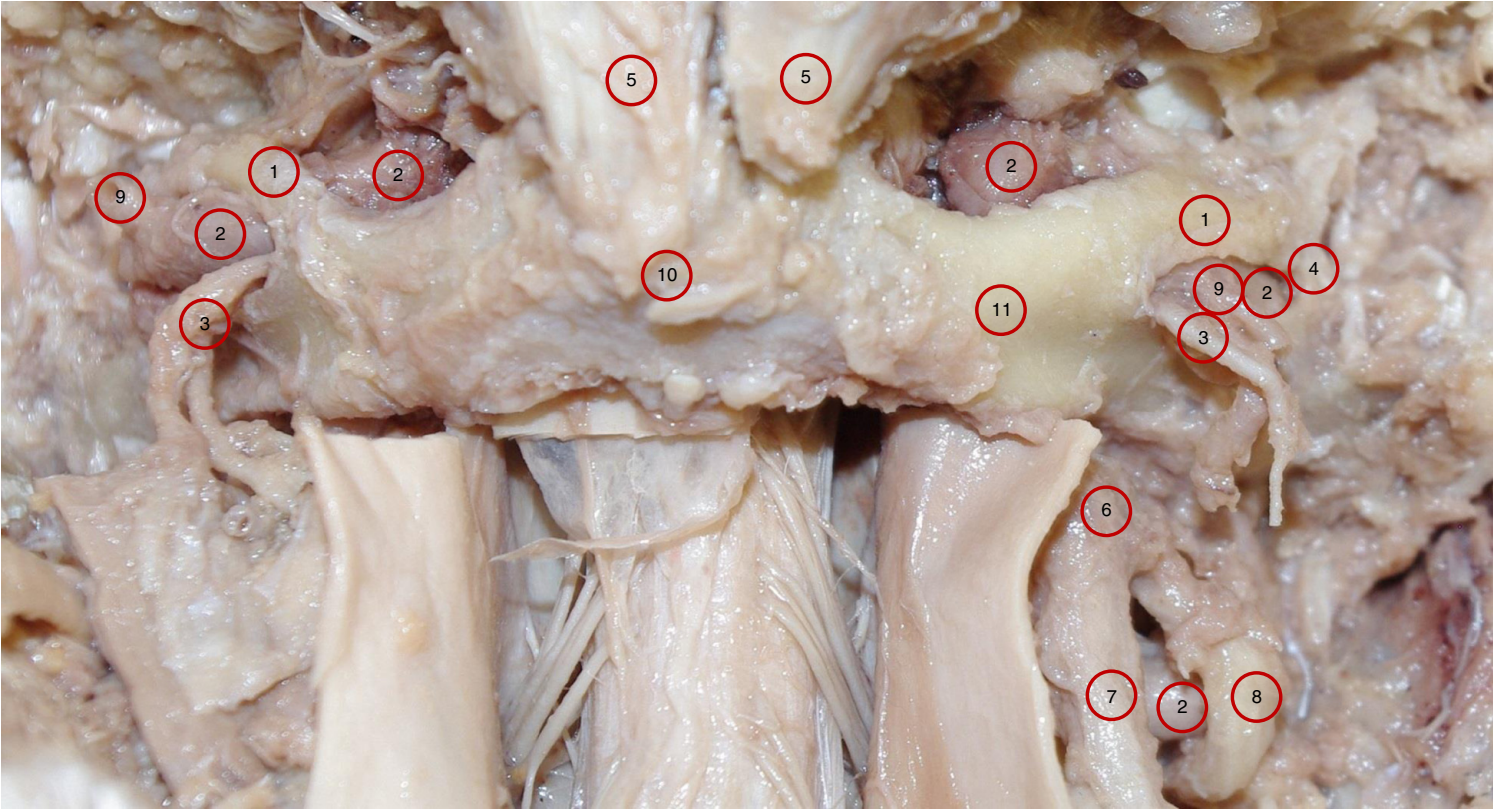
Photograph of specimen A shows a posterior view of the sub-occipital region showing the atlas with symmetrical complete bilateral dorsal osseous bridges; the lateral osseofibrous bridge; and the oval opening between the two bridges. Also, the passage of VA through the tunnel formed by the dorsal and lateral bridges. The sub-occipitall nerve (C1 dorsal ramus) along with branches of vertebral artery emerging from the oval opening. 1. Dorsal bridge; 2. Vertebral artery; 3. C1 dorsal ramus; 4. Lateral bridge; 5. Rectus capitis posterior minor; 6. C2 dorsal root ganglion; 7. C2 dorsal ramus; 8. C2 ventral ramus; 9. Branches of VA; 10. Dorsal tubercle of atlas; 11. Dorsal arch of atlas

**Fig. 2:**
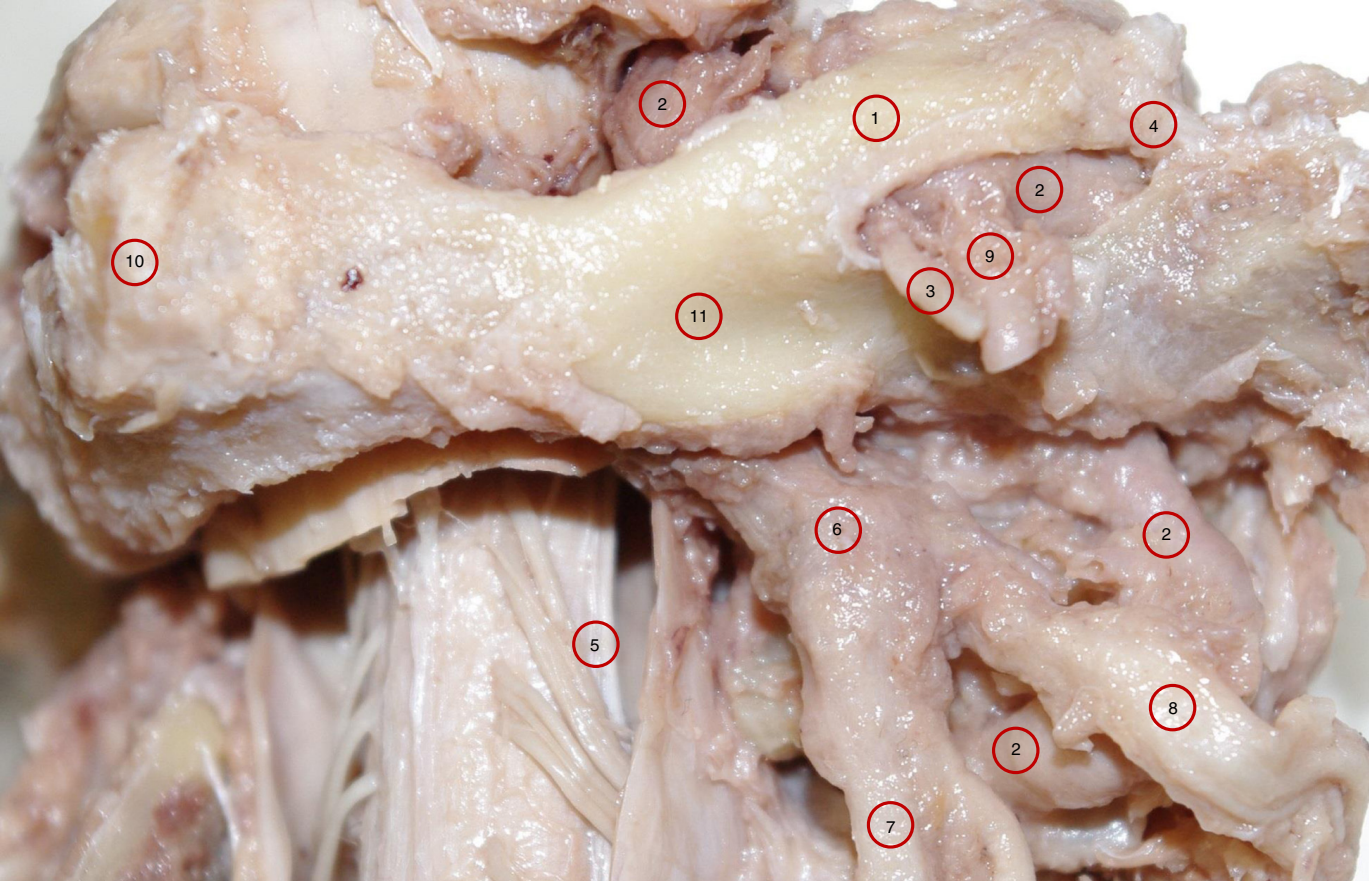
A higher magnification of specimen A of the right posterolateral aspect of sub-occipital region showing the atlas with complete dorsal and lateral bridges; and the oval opening between the two bridges. Also, the passage of vertebral artery through the tunnel formed by the posterior and lateral bridges. The sub-occipital nerve (C1 dorsal ramus) along with branches of VA emerging from the oval opening. 1. Dorsal bridge; 2. Vertebral artery; 3. C1 dorsal ramus; 4. Lateral bridge; 5. Denticulate ligament; 6. C2 dorsal root ganglion; 7. C2 dorsal ramus; 8. C2 ventral ramus; 9. Branches of VA; 10. Dorsal tubercle of atlas; 11. Dorsal arch of atlas

**Fig. 3:**
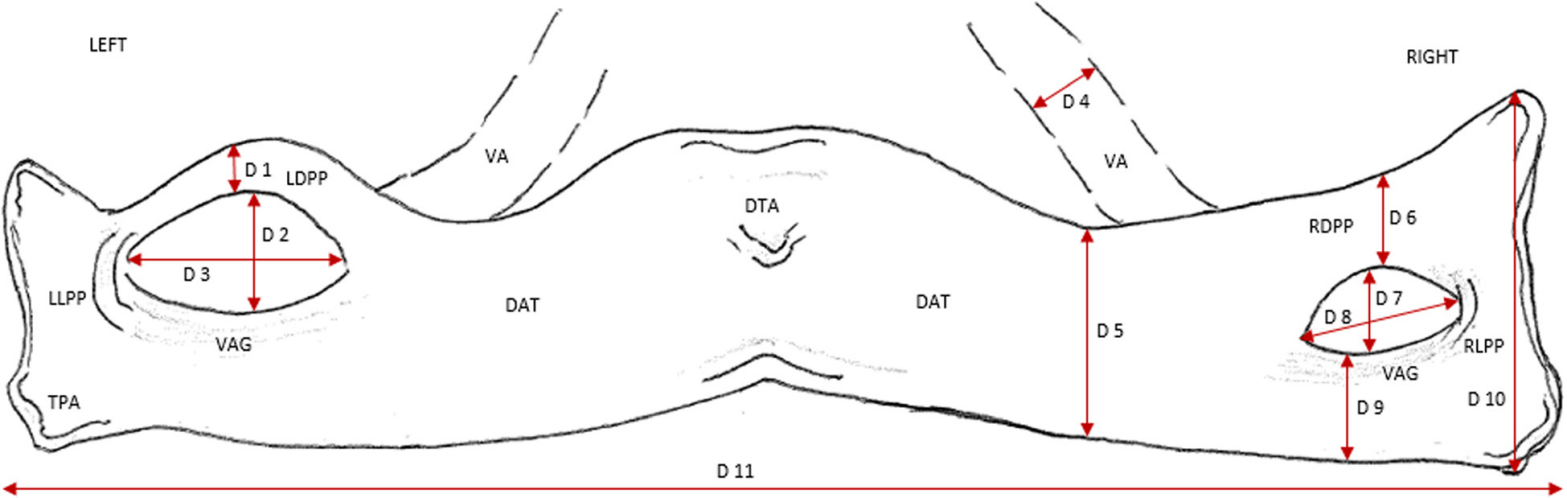
Schematic drawing corresponding to Figs. 1 and 2 showing the dimension measurements associated with the dorsal arch of the atlas: D 1 = 2.5 mm, D 4 = 3.8 mm, D 7 = 5 mm, D 10 = 18 mm, D 2 = 7.5 mm, D 5 = 10 mm, D 8 = 13 mm, D 11 = 85 mm, D 3 = 14 mm, D 6 = 5 mm, D 9 = 6 mm. LDPP = Left dorsal ponticulus posticus; LLPP = Left lateral ponsiculus posticus; RDPP = Right dorsal ponticulus posticus; RLPP = Right lateral ponticulus posticus; DAT = Dorsal arch of atlas; DTA = Dorsal tubercle of atlas; VAG = Vertebral artery groove; VA = Vertebral artery

**Fig. 4:**
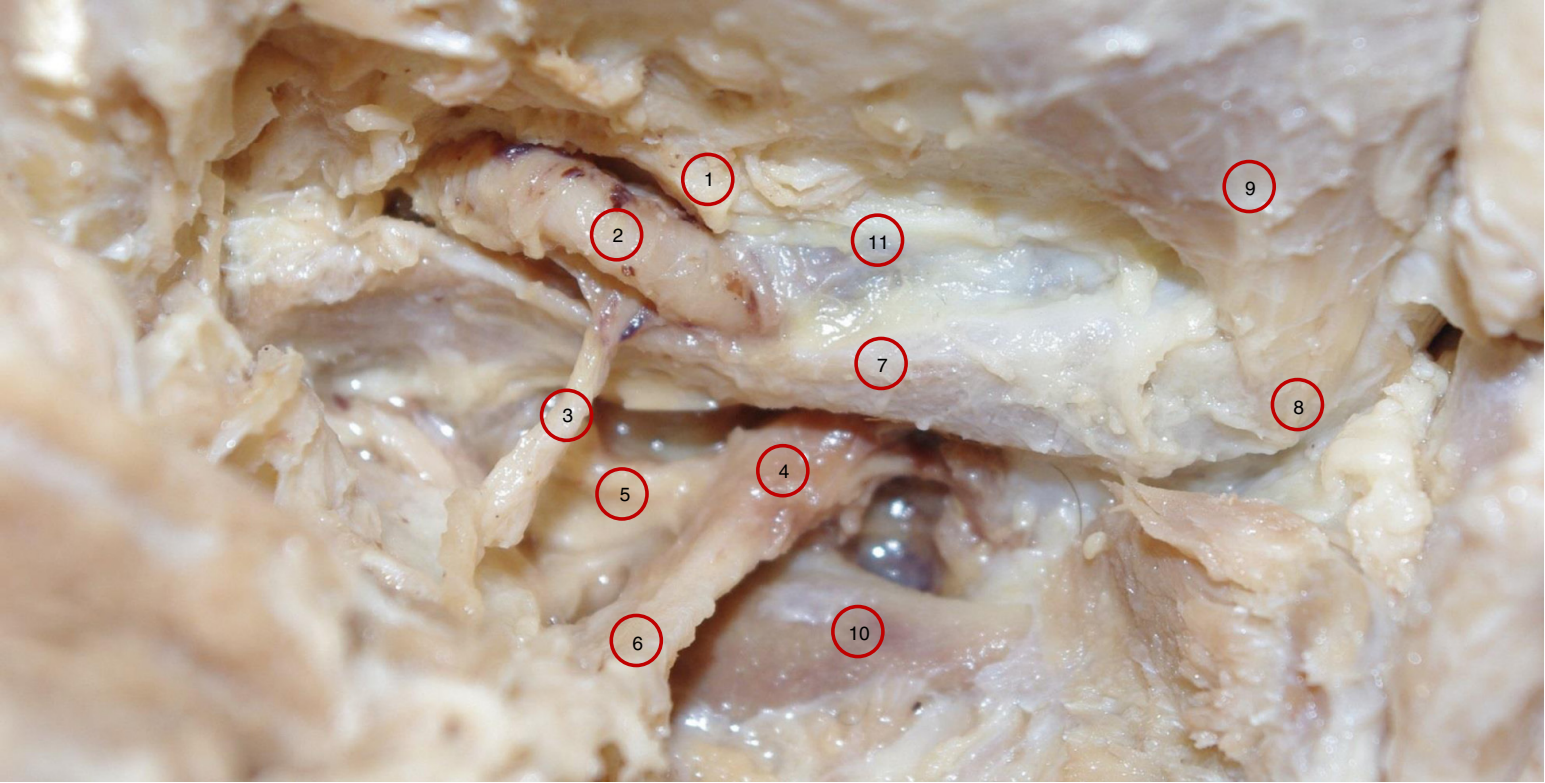
A photograph of a specimen B shows a left posterolateral view of the sub-occipital region showing vertebral artery passage from lateral to medial under the oblique inferior boarder of the atlanto-occipital membrane. This part of atlanto-occipital membrane extends from the posteromedial aspect of the superior articular process of the atlas to the medial boarder of vertebral artery groove. It is this oblique ligament that ossifies and forms the posterior complete or incomplete arcuate foramen. Note this specimen has a partial ossification of the superior aspect of the atlanto-occipital membrane. 1. Oblique part of atlanto-occipital membrane with partial ossification superiorly; 2. Vertebral artery; 3. C1 dorsal ramus; 4. C2 Dorsal root ganglia; 5. C2 Ventral Ramus; 6. C2 dorsal ramus; 7. Dorsal arch of atlas; 8. Dorsal tubercle of atlas; 9. Rectus Capitis Posterior Minor; 10. Lamina of axis; 11. Atlanto-occipital membrane

**Fig. 5:**
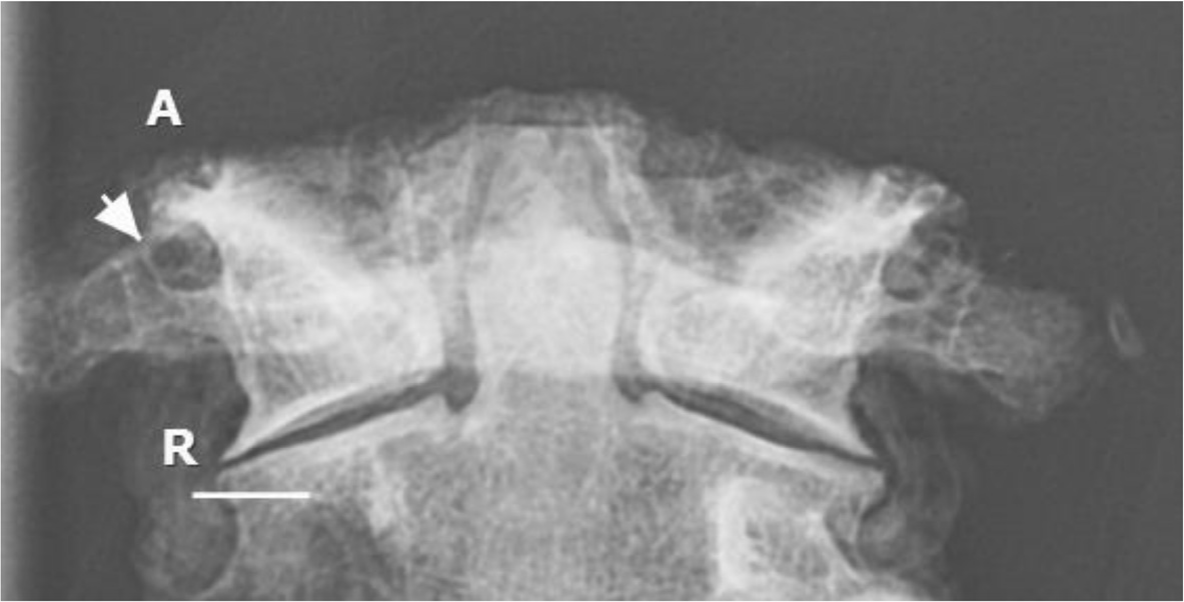
Frontal radiographic view of C1 and C2 vertebrae demonstrating complete lateral ponticle (*arrow*) on right and incomplete on the left. Linear bar represent 10 mm

**Fig. 6:**
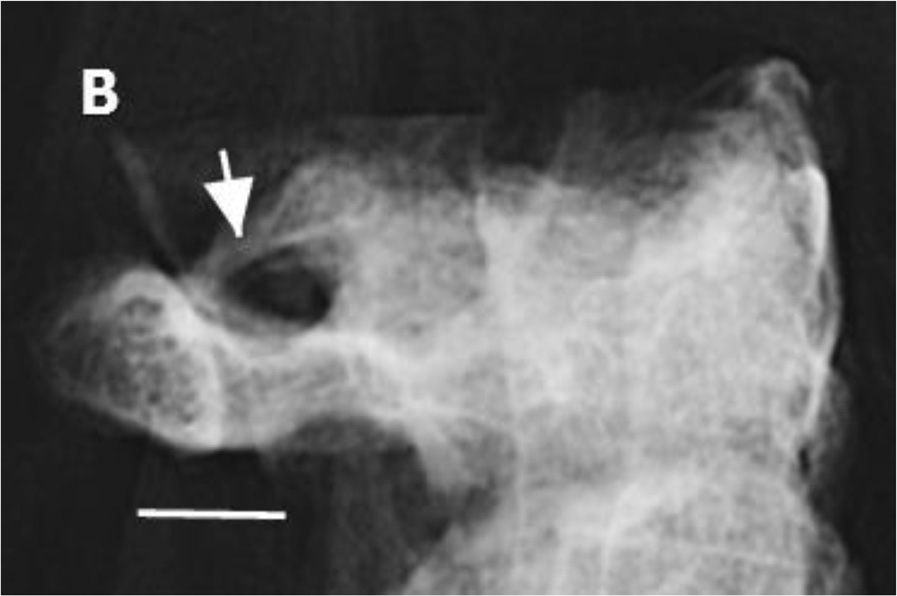
Lateral radiographic view of C1 and C2 vertebrae demonstrating complete ossification of membrane (*arrow*) forming an arcuate foramen. Linear bar represent 10 mm

Prosections are used primarily in the teaching of anatomy in disciplines as varied as human medicine, chiropractic, veterinary medicine, and physical therapy. There were 40 cadavers (20 males and 20 females) available for use in teaching anatomy in the university setting. Prosections of the suboccipital region were performed in routine teaching practices to allow students to investigate the passage of VA between foramen transversarium of the Atlas and penetration of the dura mater toward the cranial cavity. One male cadaver (Specimen A) presented with the anomalies of complete bilateral arcuate foramen, although two other cadavers had partial foramen (Specimens B and C). Visual observations were preserved with digital photography and dimension measurements were recorded (Figs. 1, 2, 3 and 4). Radiographic images were obtained in the frontal, bilateral oblique and neutral lateral (right and left) positions of the prosected suboccipital region of Specimen A to compare the visual effects of this variation (Figs. 5 and 6). These procedures were done between January 15, 2014 and August 25, 2014.

## Results

Among the 40 cadavers, only one (Specimen A) showed the presence of almost symmetrical bilateral dorsal bony bridges and lateral osseo-fibrous rings. In this specimen, VAs were found passing through the lateral foramina but deep to the dorsal bony bridge, as shown in Fig. 1, and at higher magnification of the right side in Fig. 2. The detailed dissection showed only the sub-occipital nerve (C1 dorsal ramus) along with accompanying branch of the VA emerging through the oval opening between the dorsal and lateral arches (Figs. 1 and 2). VAs were found passing deep to the oval opening on both sides, they course from lateral to medial, toward the dural sheath at the C0-C1 level on their way to the cranial cavity. The opening on the left side measured 14 mm in length and 7.5 mm in height, and on the right measured 13 mm in length and 5 mm in height. Structurally, the dorsal aspect of lateral foramina, which made the lateral borders of each dorsally faced oval opening, were osseo-fibrous i.e., they were not totally ossified. This border extended infero-laterally from the dorsolateral aspect of the C1 superior articular process to the dorsal root of the C1 transverse process. The oval windows’ medial borders were bony. These bony bridges originated from the dorsal aspect of the C1 superior articular process and fused to the C1 dorsal arch (Figs. 1 and 2). The bony bridges were the same length on both sides, but twice as thick (5 mm) on the right side as the left side (2.5 mm). As a result, the dorsal opening on the right side (5 mm) was narrower than the left side (7.5 mm). Figure 3 demonstrates the anatomical relationship with dimension measurements of the dorsal and lateral foramina.

The photographic image of Specimen A showed the VAs were intimately attached to the ossified bridges as they passed deep to the lateral and medial borders of the oval opening on both sides. Branches of VAs along with sub-occipital nerves emerged through the oval openings (Figs. 1 and 2). For comparison, the normal membranes were demonstrated in Specimens B and C, except for a partial superior ossification of the inferior oblique part of the atlanto-occipital membrane (Specimen B is shown in Fig. 4, Specimen C is not shown).

The frontal radiographic image corresponded to an AP open mouth projection (Fig. 5). Radiographic findings included bilateral lateral ponticle formations which consist of ossification in the oblique part of the atlanto-occipital membrane passing laterally from the supero-lateral aspect of the atlas lateral mass to the transverse process. This appeared as a thicker ossific band on the right with a thinner, and possibly incomplete, ossific band on the left. The lateral image revealed arcuate foramen with complete ossification of the oblique portion of the atlanto-occipital membrane bridging the posterior lateral mass and the C1 dorsal arch bilaterally (Fig. 6).

## Discussion

Specimen A showed a unique type of variation of the arcuate foramen anomaly related to the passage of the VA in the sub-occipital region. First, the VA passed from lateral to medial deep through a tunnel behind the oval windows bilaterally. Then, as it passed through the tunnel, the VA gave rise to branches which accompanied the sub-occipital nerve as it emerged dorsally through the oval window to supply the sub-occipital muscles. For comparison, the literature has provided other descriptions of VA passing through the arcuate foramina as well as a description of the normal course and position of VAs in the neck and sub-occipital region [1–8]. The two VAs, as commonly described, were found to originate from the first part of the subclavian artery and then ascend through the transverse foramina of six cervical vertebrae (C6 to C1). In the sub-occipital region, they were found to wind posterior to the atlanto-occipital joint, laying on the groove located on the superior and lateral aspect of the dorsal arch of the atlas on their way to the cranium through the atlanto-occipital membrane then through the dorsolateral aspect of the spinal dural sheath at C0-C1 level and foramen magnum. Specimen B and C demonstrated similar partial ossification of the superior aspect of the membrane. (Fig. 4 Specimen B, Specimen C is not shown)

The height and the length of the dorsal arcuate foramen in Specimen A were similar to previous studies. In our study, Specimen A measurements of the dorsal arcuate foramen included Length (14 mm Left, 13 mm Right) and Height (7.5 mm Left, 5 mm Right). In comparison, Krishnamurthy et al. found a Mean Vertical Height of 6.57 mm Left (range = 5.24 to 7.36 mm) and 6.52 mm Right (range = 6 - 6.90 mm). Krishnamurthy et al. also reported Mean Length was 7.16 mm Left (range = 5.28 - 9.56 mm) and 9.99 mm Right (range = 9.35 - 10.4 mm) [13]. Specimen A has longer length measurements because of the finding of the double foramina, similar to Tubbs et al. [5]. Tubbs et al. found both lateral and dorsal complete arcuate foramina of which the dorsal arch measured 12 mm in length. For surgeons, there is a discussion in the literature concerning surgical screw placement in the C1 lateral mass through the dorsal arch in the presence of arcuate foramen [15–19]. Of specific concern is how the false appearance of a widened dorsolateral arch would impact surgical decision for screw placement. We found the thickness of the dorsolateral aspect of the atlas arch in our specimen measured 6 mm on each side. Lee et al. reported that they found a measurement greater than 5 mm in only 13.7 % of their specimens with the average measurement only 4.13 mm. [19]. The significance of the measurement for surgical consideration is that 5 mm is the minimum requirement to safely pass a 3.5 mm screw via the C1 dorsolateral arch without injuring the VA, C2 dorsal root ganglion, C1 and C2 spinal nerves during surgical fixation of C1-C2 vertebrae (Figs. 1, 2 and 3).

There are clinical risks to consider with manual therapies with regard to whether arcuate foramina compresses the VA as it passes beneath the bony bridge which could lead to neurological conditions e.g. Vertebro Basilar Arterial Insufficiency (VBAI) [3, 25]. Mitchell and Vanitha concluded that VAs are in danger of compressive pressure resulting in stenosis from hyperextension of the head or manual pressure on the region, especially in the presence of arcuate foramen, during cervical manual manipulation [8, 25]. However Haynes, in 2005, found there was no risk of stenosis using a Doppler examination [26]. In 2001, Cushing et al. reported clinical findings that arcuate foramen caused increased incidence of VA dissection due to tethering within the arcuate foramina following traumatic events, especially with neck rotation [9]. Clinical assessment of classic signs and symptoms of VBAI should be evaluated in pre-screening procedures prior to manipulative therapies [20–22].

Our specimen showed the VAs have intimately adhered to the lateral and dorsal bridges as they passed through but were not compressed. In consideration of the risk of tethering and screw placement, it is important to include advanced imaging when the arcuate foramen is found on x-ray, especially in trauma cases. In 2014, Todd et al. reported on adverse events related to chiropractic care for children and infants but did not include arcuate foramen as an underlying pathology or increased risk for performing manual cervical manipulations [27].

In our radiographic study, we found that the arcuate foramen appeared as a ring-like structure on the lateral cervical view. However, when correlated with the true anatomical structure on the cadaveric specimen, it is not a simple ring-like foramen but it is instead an actual tunnel transmitting the vertebral vessels along with first cervical spinal nerve. No stenosis was found along the course of VAs on either side. Therefore, in such a variation of the anomaly, the vertebral artery is in a protected position rather than at risk of any physical pressures. The lateral ponticle has been reported as anatomically visible on 3 % of cervical radiographs and was present on this specimen, which identified the location of the artery on its course in a posterior - medial direction. According to Yocham and Rowe, no clinical relevance has been found to the radiographic finding of lateral ponticle [28].

## Conclusion

Arcuate foramina have been shown to be commonly found highly variable anomalies, even in the same individual with a bilateral condition. It is possible for the VAs to be compressed by the arcuate foramina. However, based on our findings, the presence of such hard bridges over the VA may provide protection from compressive forces. Since the presence of these bridges may increase the incidence of VA dissection, it is therefore clinically significant for manual therapists and chiropractors. Pre-screening clinical assessment of classic signs and symptoms of VBAI is very important. For cervical manual manipulative therapy, evaluation for the presence of partial or complete arcuate foramen is recommended with radiographic imaging. In trauma cases, advanced imaging is highly recommended. For surgical cases, since arcuate foramen is not a rare anomaly, careful evaluation of dorsal-lateral arch thickness is necessary for reducing the risk of VA, C2 dorsal root ganglion, C1 and C2 spinal nerve injuries from screw placement.

## Consent

Cadavers are used in teaching and research with donor consent, which is kept on record in the anatomy department.
